# Prevalence, distribution characteristic and risk factors of lumbar vertebral axial rotation in patients with lumbar disc herniation: a retrospective study

**DOI:** 10.1038/s41598-024-55826-8

**Published:** 2024-04-04

**Authors:** Shixian Zhao, Zhou Yao, Qiushi Wang, Peipei Huang, Zhipeng Tu, Fang Xie, Bin Ye, Yachao Ma, Zhe Wang, Zhuojing Luo, Xueyu Hu

**Affiliations:** Department of Orthopedics, Xijing Hospital, Air Force Medical University, No. 127 West Changle Road, Xi’an, 710032 Shaanxi Province China

**Keywords:** Vertebral axial rotation (VAR), Lumbar disc herniation (LDH), Range of motion (ROM), Lumbar spine, Nash–Moe, Spinal biomechanics, Anatomy, Diseases, Medical research, Risk factors, Signs and symptoms

## Abstract

This retrospective study aimed to investigate the impact of lumbar disc herniation (LDH) on vertebral axial rotation (VAR) in the lumbar spine, focusing on both close and distant neighboring vertebrae. A total of 516 patients with LDH and an equal number of healthy individuals were included in the study, matched for age and gender. The degree of axial rotation for each lumbar spine vertebra was assessed using the Nash–Moe index. The results revealed that the prevalence of VAR in the lumbar spine was significantly higher in the LDH group compared to the Control group (65.7% vs 46.7%, *P* < 0.001). Among the LDH group, the L2 vertebra had the highest frequency of VAR (49.5%), followed by L1 (45.1%), and then L3 to L5 (33.6%, 8.9%, 3.1%, respectively). A similar pattern was observed in the Control group (L2, 39.8%; L1, 34.6%; L3, 23.2%; L4, 3.1%; L5, 0.8%). Furthermore, the study found that disc herniation was associated with a higher incidence of VAR not only in close neighboring vertebrae but also in distant neighboring vertebrae. This indicates that the biomechanical influence of LDH extends beyond just the immediate adjacent vertebrae. To identify potential risk factors for VAR in LDH patients, multivariate analysis was performed. The results revealed that age was an independent risk factor for VAR (OR 1.022, 95% CI [1.011, 1.034], *P* < 0.001). However, the duration of symptoms and presence of back pain were not found to be significant risk factors for VAR.

## Introduction

Vertebral axial rotation (VAR) is a crucial factor in evaluating spinal stability^1^. Studies^2–6^ have shown that VAR plays a significant role in scoliosis, such as adolescent idiopathic scoliosis (AIS) and degenerative lumbar scoliosis (DLS). A longitudinal study which track the progression of spinal deformity reported that in grade II and III cases by Nash and Moe’s method, Cobb angle increased by more than 10° in patients with adult spinal deformity who had follow-up for at least 10 years^7,8^. A retrospective research showed patients with greater apical axial vertebral rotation had significantly worse Oswestry Disability Index and more low back pain in scoliosis^9^. Furthermore, for patients requiring operative treatment, preoperative assessment of the VAR of the apex is necessary for planning surgical strategies and instrument placement^10,11^.

However, the VAR in other lumbar degenerative diseases, especially lumbar disc herniation (LDH) is still unclear^12^. Therefore it is very necessary to identify the VAR in LDH. Previously, many ex vivo studies explored the relationship between VAR and disc degeneration, have got partial knowledge. Some biomechanical researches with specimens suggested that intervertebral axial rotation may contribute to disc herniation by placing additional strain on the annular fiber^13,14^. Conversely, other biomechanical researches investigated the impact of disc degeneration on lumbar kinematics, specifically the range of motion (ROM), using various methodologies. Mimura et al.^15^ performed a biomechanical test to assess the flexibility of 47 discs from 12 intact lumbar spine specimens without preload. They discovered that disc degeneration leads to an increase in ROM during axial rotation. Fujiwara et al.^16^ conducted a study using a larger sample of 110 lumbar motion segments from 44 human lumbar spines and applied moments ranging from 0 to 6.6 Nm. Their findings indicated that both males and females experience increased axial rotational motion as a result of disc degeneration. Furthermore, a study with a substantial in vitro data set supported the idea that intervertebral disc degeneration is linked to a decrease in axial rotation stability^17^.

Despite these results being relatively consistent, most of the above mentioned studies relied on ex vivo assessment. There was a relatively insufficient number of in vivo investigations, which could reflect the true state of VAR in patients with LDH. Hence, an in vivo study was designed. In this study, after age and gender matched, we retrospectively collected 516 patients with LDH and utilized X-ray films to evaluate VAR. The primary objective was to identify the overall characteristics of VAR in the lumbar spine of patients with LDH, whilst the secondary objective was to explore any potentially significant clinical factors associated with VAR in LDH. As far as we were concerned, the comprehensive understanding of VAR in patients with LDH would be helpful for assessment of spinal stability, surgical strategies planning and instrument placement.

## Result

In this retrospective study, we identified a total of 2564 inpatients with LDH (L4-S1) and 2590 outpatients between March 2011 and September 2021. After matching for age and gender, there were 516 subjects in each group (LDH group and Control group). The demographic and clinical characteristics between the two groups showed no significant differences (Table 1). However, there were slightly more patients with osteoporosis in the LDH group compared to the Control group (116 vs 92, *P* = 0.07).

**Table 1:** Demographic data and clinical characteristics.

	LDH Group (n = 516)	Control Group (n = 516)	*P* value
Age (years)	52.22 ± 11.72	52.22 ± 11.72	1
Gender, n (%)			1
Male	308 (59.7)	308 (59.7)	
Female	208 (40.3)	208 (40.3)	
BMI (kg/m^2^)	23.88 ± 3.47	24.34 ± 6.45	0.18
Comorbidity			
Hypertension	85	72	0.29
Diabetes	28	19	0.23
Osteoporosis	116	92	0.07
Others	86	78	0.55
LDD grade	3.28 ± 0.55	–	
Level of disc herniation			
L4/5	263	–	–
L5/S1	148	–	–
L4/S1	105	–	–

LDD grade was presented by the mean value of Pfirrmann scoring from L1 to S1.

*BMI* body mass index.

All patients with LDH underwent MRI, and the average lumbar disc degeneration (LDD) grade was 3.28. In contrast, only 43 subjects in the Control group had MRI records. After matching for age and gender, the LDH group had a higher LDD grade compared to the Control group (3.43 ± 0.67 vs 2.79 ± 0.78, *P* < 0.001).

The inter-observer and intra-observer variability in this study demonstrated very good consistency. The variability between observers for both VAR (kappa = 0.85, *P* < 0.001) and Pfirrmann scoring (kappa = 0.78, *P* = 0.02) was high, indicating good agreement. Similarly, the intra-observer variability for VAR (kappa = 0.96, *P* < 0.001) and Pfirrmann scoring (kappa = 0.88, *P* = 0.01) showed excellent consistency.

### Prevalence of VAR in lumbar spine

The study found that the overall prevalence of VAR in the entire lumbar spine was 65.7% in the group with lumbar disc herniation (LDH), while it was 46.7% in the Control group. This difference was statistically significant (*P* < 0.001), indicating a higher prevalence of VAR in the LDH group.

Furthermore, when comparing the prevalence of VAR at each specific lumbar level, it was found that the LDH group had significantly higher rates compared to the Control group. At the L1 level, the prevalence of VAR was 49.8% in the LDH group compared to 34.5% in the control group (*P* < 0.001). Similarly, at the L2 level, the prevalence was 54.7% in the LDH group and 39.5% in the Control group (*P* < 0.001). The LDH group also showed significantly higher rates of VAR at the L3 (37.2% vs 23.3%, *P* < 0.001), L4 (10.7% vs 3.5%, *P* < 0.001), and L5 (3.5% vs 0.8%, *P* = 0.03) levels. Additionally, the study found that the prevalence of VAR was significantly higher in both the close and distant neighboring vertebrae of the LDH group compared to the Control group. These findings highlight the association between VAR and lumbar disc herniation, suggesting that the presence of VAR may contribute to the development of LDH.

To address potential confounding factors, the LDD grade was matched 1:1 in the two groups, resulting in 27 patients in each group. The total prevalence of VAR differed significantly between the groups (74.1% vs 44.4%, *P* = 0.03). When conducting subgroup analysis of VAR in patients with osteoporosis, there was a significant difference between the groups (63.8% vs 51.1%, *P* = 0.04) (Supplementary Table S1). Similarly, subgroup analysis of VAR in patients without osteoporosis showed a significant difference (47.8% vs 31.8%, *P* = 0.02) (Supplementary Table S2).

Based on our findings, there was no significant difference in the prevalence of VAR between males and females in both the LDH group and the Control group. In the LDH group, the prevalence of VAR was found to be 65.6% in males and 65.9% in females (*P* = 0.947). When comparing specific vertebral levels, there was no significant difference observed for L1 (49.7% vs 50.0%, *P* = 0.942), L2 (55.5% vs 53.4%, *P* = 0.63), L3 (36.7% vs 38.0%, *P* = 0.766), L4 (10.7% vs 10.6%, *P* = 0.96), and L5 (3.3% vs 3.9%, *P* = 0.716). Similarly, in the Control group, the prevalence of VAR was not significantly different between males and females. The total prevalence was 45.8% in males and 48.1% in females (*P* = 0.608). When examining specific vertebral levels, there were no significant gender differences observed for L1 (33.4% vs 36.1%, *P* = 0.54), L2 (39.3% vs 40.4%, *P* = 0.802), L3 (21.8% vs 25.5%, *P* = 0.326), L4 (3.9% vs 2.9%, *P* = 0.539), and L5 (0% vs 1.9%, *P* = 0.608). These findings indicate that gender does not appear to influence the prevalence of VAR in either the LDH group or the Control group.

### Distribution characteristic of VAR in lumbar spine

**Figure 1:**
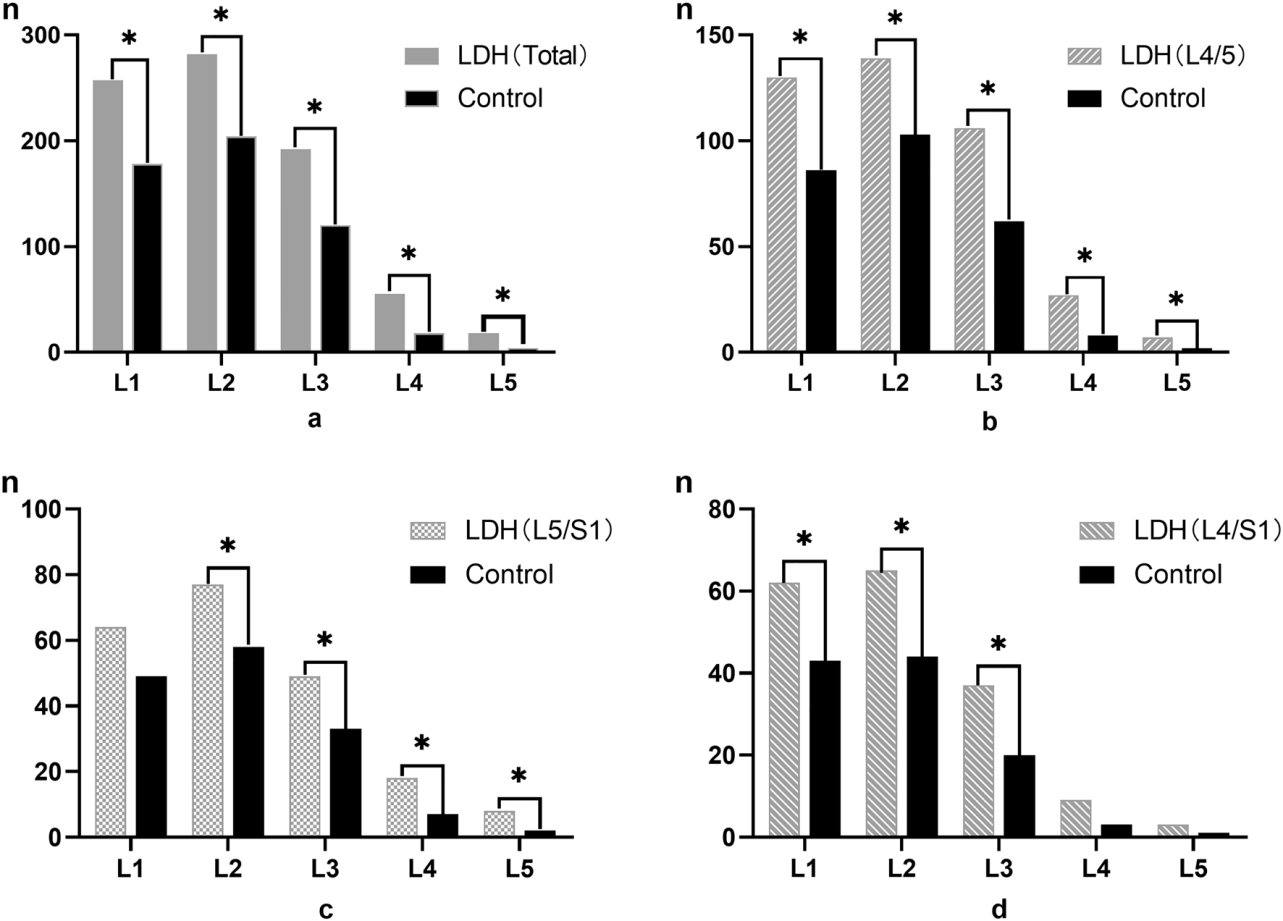
The distribution of vertebral axial rotation (VAR) in LDH and Control group. (**a**)The overall distribution of VAR in all of the patients. (**b**) The distribution of VAR in L4-5 subgroup. (**c**) The distribution of VAR in L5-S1 subgroup. (**d**) The distribution of VAR in L4-S1 subgroup. *P < 0.05.

In the LDH group, we observed that the highest prevalence of vertebral axial rotation (VAR) was found in the L2 vertebra at 49.5%. This was followed by the L1 vertebra at 45.1%. As we moved down the lumbar spine from L3 to L5, the prevalence of VAR gradually decreased, with rates of 33.6%, 8.9%, and 3.1%, respectively. Similarly, the Control group showed a comparable trend in VAR prevalence. The L2 vertebra had a prevalence of 39.8%, followed by the L1 vertebra at 34.6%. Moving down the lumbar spine, the prevalence of VAR decreased at a similar rate. The L3 vertebra had a prevalence of 23.2%, while the L4 and L5 vertebrae had rates of 3.1% and 0.8%, respectively (Fig. 1a).

Subgroup analysis revealed that in cases where disc herniation occurred at the L4-L5 segment, there was a significant difference in VAR prevalence in each lumbar vertebra from L1 to L5 (*P* < 0.05) (Fig. 1b). However, when disc herniation occurred at the L5-S1 segment, the difference in VAR prevalence was evident in the L2, L3, L4, and L5 vertebrae, but not in the L1 vertebra (Fig. 1c). In cases where disc herniation occurred at the L4-S1 segment, the difference in VAR prevalence was significant in the L1, L2, and L3 vertebrae, but not in the L4 and L5 vertebrae (Fig. 1d). These findings suggest that disc herniation is not only associated with an increased prevalence of VAR in closely neighboring vertebrae but also in distant neighboring vertebrae.

Table 2 presents a comprehensive comparison of VAR in different disc herniation segments. The results show that there is no significant difference in VAR prevalence between the entire lumbar spine or among close neighboring vertebrae in the subgroups. However, there is a significant difference in VAR prevalence among distant neighboring vertebrae. Specifically, the L5-S1 subgroup demonstrates a lower prevalence of VAR compared to both the L4-L5 subgroup (35.41% vs 47.53%, *P* = 0.015) and the L4-S1 subgroup (35.41% vs 52.07%, *P* = 0.006). However, the difference in VAR prevalence between the L4-L5 subgroup and the L4-S1 subgroup is not statistically significant (47.53% vs 52.07%, *P* = 0.4).

**Table 2:** Subgroup analysis of lumbar vertebral axial rotation at different disc herniation segment.

	L4/5 (n = 263)	L5/S1 (n = 148)	L4/S1 (n = 105)	*P* value	*P* value (L4/5 *vs* L5/S1)	*P* value (L5/S1 *vs* L4/S1)	*P* value (L4/5 *vs* L4/S1)
Total^a^	409 (31.10%)	216 (29.19%)	176 (33.52%)	0.766	0.653	0.468	0.689
Close neighboring vertebrae^a^	34 (6.46%)	8 (5.41%)	12 (5.71%)	0.901	0.666	0.916	0.789
Distant neighboring vertebrae^a^	375 (47.53%)	208 (35.41%)	164 (52.07%)	0.012^b^	0.015^c^	0.006^c^	0.400

^a^The number and percent of vertebrae axial rotation.

^b^*P* value < 0.05; The pairwise comparison among these three subgroups was performed by Tukey’s post hoc test, and corrected α was 0.017.

^c^*P* value < 0.017.

### The risk factors for VAR in patients with LDH

The LDH group was divided into two groups based on the presence or absence of VAR (Rotation group and Non-rotation group). It was found that all vertebrae in the Non-rotation group had a Nash–Moe index of 0, while in the Rotation group, at least one vertebra had a Nash–Moe index greater than grade 1.

**Figure 2:**
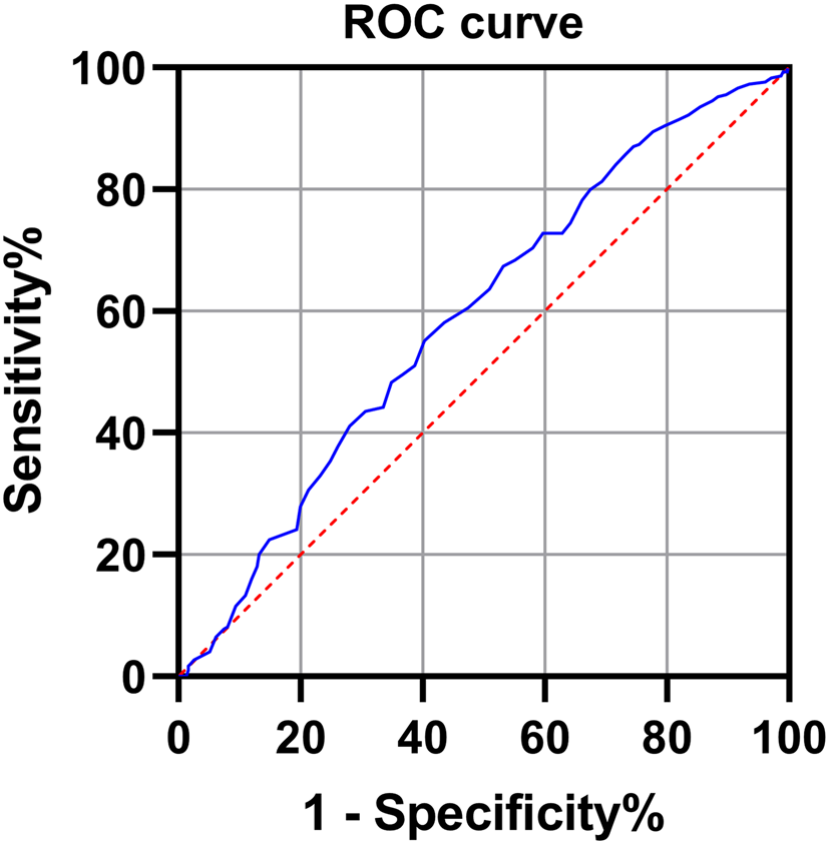
Age was associated with increased incidence of VAR in LDH (AUC = 0.589). The cutoff point was 43.5 years.

The results of univariate analysis showed that the mean age in the Rotation group was significantly higher than that in the Non-rotation group (*P* < 0.001). There were no significant differences in gender, BMI, smoking and drinking status, or clinical medical comorbidities between the two groups, except for osteoporosis (*P* = 0.003). Additionally, there were no statistical differences in VAS back pain and symptom duration between the two groups (Table 3). Age and osteoporosis were identified as potential predictive variables for VAR. Stepwise forward logistic regression analysis revealed that age was the only risk factor for VAR (OR 1.022, 95%CI [1.011, 1.034], *P* < 0.001) (Table 4). Furthermore, ROC curve indicated that age is associated with incidence of VAR in patients with LDH (AUC = 0.589, cutoff point was 43.5 years) (Fig. 2). The linear correlation analysis demonstrated a positive correlation between VAR and age (r = 0.083, *P* = 0.05). Similarly, the LDH group showed similar results regarding the correlation between age and VAR (r = 0.15, *P* < 0.001) (Supplementary Fig. S1).

**Table 3:** Difference of characteristics in LDH group with and without VAR.

	Rotation group (n = 265)	Non-rotation group (n = 251)	*P* value
Age, years^a^	46.7 (14.9)	42.3 (13.7)	< 0.001^c^
Gender, n (%)			0.615
Male	133 (50.2)	131 (52.2)	
Female	132 (49.8)	120 (47.8)	
BMI, kg/m^2a^	24.1 (3.5)	23.7 (3.4)	0.138
Hypertension	50	35	0.154
Diabetes	16	12	0.565
Osteoporosis	74	42	0.003^c^
Smoking	47	37	0.404
Drinking	17	16	0.985
VAS Back^b^	4 (3)	4 (5)	0.076
Symptom duration^b^	36 (76)	24 (54)	0.128

*BMI* body mass index.

^a^Mean and standard deviation.

^b^Median and interquartile range.

^c^*P* < 0.05.

**Table 4:** Multivariate logistic regression analysis of risk factors for vertebral axial rotation in patients with lumbar disc herniation.

	OR	95% CI		*P* value
		Lower	Upper	
Age, per year	1.022	1.011	1.034	< 0.001

*OR* odds ratio, *CI* confidence interval.

## Discussion

VAR, or vertebral axial rotation, is a common occurrence in lumbar degenerative disease and is indicative of instability in the motion segment in the transverse plane. With the increasing use of robot-assisted pedicle screw placement, VAR has become an important measurement index. Previous studies have shed light on the relationship between VAR and disc herniation in cases of LDH. However, there are still several unresolved issues that need further investigation. Therefore, an in vivo investigation is crucial and necessary to supplement the existing knowledge in this area.

In the current study, we observed that VAR exists not only in LDH patients, but also in healthy individuals. This findings provided more evidence for the pre-existing VAR in healthy individuals, which aligns with previous studies^18^. Besides, our results suggested a higher overall prevalence of VAR in LDH patients compared to healthy individuals (65.7% vs 46.7%, *P* < 0.001). More importantly, the results remained statistically significant even after adjusting for several potential confounding factors, including osteoporosis and lumbar disc degeneration (LDD) grade. This suggests that VAR is a distinct characteristic observed in patients with LDH. According to the clinical practice and previous researches, the VAR may be a secondary change in LDH. To investigate the potential impact of disc herniation on VAR, various methods for measuring the range of motion (ROM) of intervertebral axial rotation and different classification systems for assessing disc degeneration have been utilized. Mimura et al. and Fujiwara et al. conducted biomechanical tests on lumbar motion segments from human cadavers, with or without preload. Their findings consistently demonstrated that disc degeneration increased the axial rotational motion of each lumbar segment^15,16^. Additionally, several other biomechanical and imaging studies have reported positive correlations between Pfirrmann grade and axial rotation ROM^5,19,20^. Moreover, research by Haughton and Krismer indicated that the presence of annulus fibrosus tears or high-intensity zones (HIZs) significantly contributed to axial rotation instability^21,22^. Our study’s results indicated that LDH is associated with a higher prevalence of VAR compared to healthy individuals, which is consistent with earlier research findings. This further supports the notion that disc herniation has an impact on VAR.

In this study, we observed a specific distribution trend of VAR in the lumbar spine, with the highest prevalence of VAR found in the L2 vertebra (49.5%), followed by the L1 vertebra (45.1%), and then a gradual decrease in prevalence from L3 to L5 (33.6%, 8.9%, 3.1%). One possible explanation for this trend is that the L1 and L2 vertebrae are located at the thoracolumbar junction, where they are not constrained by the thoracic spine or as much by surrounding muscles and ligaments. This may result in a larger range of motion (ROM) in these vertebrae, leading to a higher prevalence of VAR. On the other hand, as the lumbar segments go down, the lower vertebrae have stronger muscles and a greater number of ligaments attached to them. This increased muscular support and ligamentous stability may minimize movement and reduce the prevalence of VAR in these lower vertebrae. Furthermore, the facet joints in the upper lumbar segments of a normal spine are oriented more sagittally (front-to-back) compared to the facet joints in the lower segments. This difference in facet joint orientation may also contribute to the VAR distribution pattern observed in our study^23^. This special orientation enables upper segments to resist axial rotations more effectively^24^, which could explain why the prevalence of VAR is higher in the L2 vertebra compared to the L1 vertebra.

Our findings demonstrate, for the first time to our knowledge, that disc herniation not only impacts the close neighboring vertebrae but also increases the prevalence of VAR in distant neighboring vertebrae. This suggests the existence of an underlying force conduction mechanism in the lumbar spine. There are several reasons to support this inference. Firstly, regardless of the specific segment of disc herniation, we observed a higher prevalence of VAR in distant neighboring vertebrae compared to healthy individuals. This suggests that the influence of disc herniation extends beyond the immediate neighboring vertebrae. Furthermore, when disc herniation occurred at the L4-5 segment (either L4-5 or L4-S1 subgroup), there was a significant difference in VAR observed in distant neighboring vertebrae up to the L1 vertebra. However, when disc herniation involved the L5-S1 segment, the significant difference in VAR was observed only up to the L2 vertebra. The reasons for this discrepancy between the two disc herniation segments require further investigation. It is worth noting that in cases where disc herniation involved double levels at the L4-S1 segment, the close neighboring vertebrae did not show a significant difference in VAR compared to healthy individuals. This may indicate that factors other than disc herniation, such as the interaction between adjacent discs or individual variations, play a role in determining the impact on VAR. Further research is needed to fully understand and clarify these potential reasons for the observed variations in VAR prevalence in different segments of disc herniation.

In this study, we aimed to investigate the possible factors associated with VAR in LDH. Our analysis firstly revealed that age is an independent risk factor for VAR, as indicated by the results of our multivariate logistic regression analysis (OR 1.022, 95%CI [1.011, 1.034], *P* < 0.001). The relationship between age and VAR has been identified by many researches. A prior systematic review and meta-analysis demonstrated the decline in range of motion (ROM) in axial rotation with aging^25^. Another previous research have demonstrated pre-existent VAR in normal spine, and the rotational pattern seemed more pronounced in the adolescent age (10–16 years old) than infantiles (0–3 years old) and juveniles (4–9 years old)^26^. Besides, another study measured range of movement in 100 healthy subjects aged 20 to 60+ years, and the results showed the stability in axial rotation decline with aging^27^. In our study, we reviewed subjects aged more than 18 years old, and the linear correlation analysis showed VAR positively correlated with age in subjects with or without LDH (Supplementary Fig. S1). It could be inferred that age was a direct related factor to VAR. Osteoporosis is a common condition characterized by reduced bone quality and has been found to have a positive association with age^28^. However, in our final model, we had to exclude osteoporosis due to the presence of multicollinearity. Interestingly, a previous investigation assessed vertebral bone densities in cadavers using CT scans and found a positive correlation with lumbar spine axial stability. It is important to note that this study did not consider the age of the cadaveric lumbar spine^29^. One interesting finding in our study is that VAS back pain scores and symptom duration were not found to be relevant factors for VAR in LDH. This contrasts with previous studies that have reported contradictory results. Some researchers have suggested that vertebral axial rotational instability is associated with low back pain (LBP). For example, Blankenbaker et al. used discography to measure axial rotation in sixteen patients with and without concordant pain. Their study showed that the axial rotation of the L3-L4 and L4-L5 discs increased in patients with concordant pain^30^. Similarly, Basques et al. recruited ninety-nine volunteers, including both asymptomatic individuals and those with LBP, and found that LBP was associated with greater axial rotation at the L4/L5 region^31^. It is worth noting that our study examined the VAR of the entire lumbar spine, while these previous studies focused specifically on the intervertebral axial rotation of the vertebrae involved in LBP. This difference in focus may contribute to the discrepancies observed between our findings and those of previous investigations. In our opinion, the exploration of risk factors would be helpful for prediction of progression of VAR in patients with LDH.

This research has several limitations. Firstly, our study was conducted at a single center and had a retrospective design. This introduces the possibility of selection bias and limits the generalizability of our findings. To overcome these limitations, future research should consider conducting a multicenter prospective study with a larger sample size to validate our results. Secondly, in this study, assessment of VAR was based on Nash–Moe index on plain film. This may not indicate the rotation degerees as accurate as on CT scan^32^. Finally, in this study, the orientation of facet joint was not taken into consideration. Our research group found that facet tropism have a small influence on VAR in patients with LDH^33^. More rigorous research is needed to provide better knowledge about these relationships.

In conclusion, with large samples, our in vivo study has preliminarily discribed the prevalence and distribution characteristic of VAR in entire lumbar segment of LDH patients. Besides, our findings demonstrated that both close and distant neighboring vertebrae with disc herniation have a higher frequency of VAR. Additionally, age was found to be an independent risk factor for VAR in LDH. These findings would help to provide guidance for early assessment of spinal instability, predict the progression of axial rotation, and increase accuracy of instrument placement in patients with LDH.

## Materials and methods

### Ethics statements

This study was performed according to the principles of the Declaration of Helsinki of the World Medical Association. This study was approved by Ethics Committees of the Air Force Medical University. All medical records have been irrevocably anonymized, and this study is a retrospective study, so the informed consent requirement is waived. Waived consent was approved by Ethics Committees of xijing hospital, Air Force Medical University (ky20222166).

### Patient population

This study was a retrospective case–control study. Inpatient with LDH who received underwent elective lumbar surgery were screened at single center from March 2011 to September 2021. Inclusion criteria were as follows: (1) more than 18 years old; (2) MRI scanning showed single or double level herniation in L4-S1 (protrusion, extrusion or sequestered fragment) consistent with the clinical symptoms; (3) having preoperative anteroposterior radiographs of lumbar spine. Exclusion criteria were as follows: (1) patients with symptoms lasting less than 3 months; (2) those who have lumbar fracture, acute infections, active systemic infections, or infections at the surgical site; (3) prior history of lumbar surgery; (4) prior history of malignant tumors; (5) patients with low-quality preoperative X-ray images, lumbosacral transitional vertebrae, lumbar scoliosis, spondylolisthesis or spondylolysis.

In our study, the case–control group consisted of individuals without lumbar disc herniation (LDH) who were selected from the outpatient clinic and had come in for a health examination. These individuals had anteroposterior radiographs of the lumbar spine available for analysis. To minimize the influence of potential confounding factors, we used a 1:1 matching strategy to match subjects from the LDH group with individuals in the control group based on age and gender.

### Demographic and clinical data collection

In our study, the demographic data of the two groups includes sex, age, height, weight, smoking and drinking status, symptom duration, and medical comorbidities. Body mass Index (BMI) was calculated according to height and weight, and other relevant data, such as preoperative visual analogue scale (VAS) scores for low back pain (ranging from 0 to 10), were obtained from the participants’ medical records.

### Radiographic measurement

In order to assess the vertebral axial rotation (VAR) of the lumbar spine (L1-L5) on plain films, we utilized the Nash–Moe index^29^. Nash–Moe index not less than Grade 1 was defined as vertebral axial rotation (VAR), whereas Grade 0 was non-rotation. The VAR was measured by two independent observers (QSW and ZY) in a blinded fashion. If there was a discrepancy between the two readings, the senior observer’s (ZY) judgment was deemed to be final. Weighted kappa values were used to calculate the inter-observer variability (the criteria for judging the consistency strength of a kappa coefficient as follows: 0.20, bad; 0.21–0.40, fair; 0.41–0.60, acceptable; 0.61–0.80, very good; and 0.81–1.00, excellent). The primary observer (QSW) evaluated VAR again after four weeks, and weighted kappa values of intra-observer variability were determined as previously mentioned.

In our study, we evaluated the prevalence of vertebral axial rotation (VAR) at each lumbar level and estimated the overall prevalence of VAR in the lumbar spine. To analyze the effect of disc herniation on VAR of vertebrae, we categorized the neighboring vertebrae into two groups. The close neighboring vertebrae were defined as the upper and lower vertebrae closest to the disc affected by herniation. For example, in cases of disc herniation at the L4-L5 or L4-S1 segment, the close neighboring vertebrae were L4 and L5. Similarly, for disc herniation at the L5-S1 segment, L5 was designated as the close neighboring vertebra. On the other hand, the distant neighboring vertebrae encompassed the remaining vertebrae in the lumbar spine (i.e., L1, L2, L3, and in some cases L4) for patients with disc herniation at the L4-L5 or L4-S1 segment. For patients with disc herniation at the L5-S1 segment, the distant neighboring vertebrae were L1, L2, L3, and L4. By categorizing neighboring vertebrae into close and distant groups according to the site of disc herniation, we aimed to explore the potential influence of disc herniation on VAR in different regions of the lumbar spine. This allowed us to examine the association between VAR and specific disc herniation segments and neighboring vertebrae.

The lumbar magnetic resonance imaging (MRI) was conducted using a 1.5T MRI system (Siemens MAGNETOM Symphony). The MRI images were taken from the level of L1 to S1 to evaluate the severity of lumbar disc degeneration (LDD). The assessment of LDD was performed on the mid-sagittal MR images. To determine the degree of degeneration, we utilized the Pfirrmann scoring system. This system classifies the degeneration into five different grades based on the signal intensity of the nucleus pulposus and disc height.

The LDD grade was presented by the mean value of Pfirrmann scoring from L1 to S1.The inter-observer and intra-observer variability were assessed by weighted kappa values in the same way described above (The intra-observer analysis interval was 2 weeks).

### Statistical analysis

All statistical analyses were done using Statistical Package for Social Sciences for Windows version 25.0 (SPSS Company, USA). Age and BMI, which are continuous variables with a normal distribution, were expressed as mean ± SD, and VAS and symptom duration were given as median and interquartile spacing (IQR). The independent sample One-way analysis of variance (ANOVA) and nonparametric Mann–Whitney test were used to determine whether a statistically significant difference was present. To account for potential confounding factors, LDD grade was matched in the LDH and Control groups. Furthermore, the overall prevalence of VAR between the two groups was re-analyzed based on the presence or absence of osteoporosis. The LDH group was further subdivided into three subgroups based on the segment of disc herniation, and pairwise comparisons among these subgroups were conducted using Tukey’s post hoc test. To identify risk factors for VAR in LDH, multivariate logistical regression was performed by stepwise regression (Forward: LR). Receiver operating characteristic (ROC) of the curve was conducted to assess the area under the curve (AUC) of age in relation to VAR. Additionally, linear correlation analysis was performed to examine the relationship between age and VAR within the LDH and Control groups. Statistical significance was defined as a *p* value less than 0.05.

### Supplementary Information

Supplementary Information.

## Data Availability

The datasets used and/or analyzed during the current study are available from the corresponding author on reasonable request.
