# Supplementary material for: Prevalence, distribution characteristic and risk factors of lumbar vertebral axial rotation in patients with lumbar disc herniation: a retrospective study
Source: Sci Rep. 2024 Apr 4;14:7909. doi: 10.1038/s41598-024-55826-8 (PMC10995131; doi:10.1038/s41598-024-55826-8)
Supplement: Supplementary file 1 — Supplementary Information. [file 41598_2024_55826_MOESM1_ESM.pdf]

Title: Prevalence，Distribution Characteristic and Risk Factors of Lumbar Vertebral Axial Rotation in Patients with Lumbar Disc Herniation：A Retrospective Study

Authors： Shixian Zhao [†], Zhou Yao [†], Qiushi Wang [†], Peipei Huang, Zhipeng Tu, Fang Xie, Bin Ye, Yachao Ma，Zhe Wang, Zhuojing Luo[†]* and Xueyu Hu[†]*

***Correspondence:** lzjxijing@163.com; huxueyu@fmmu.edu.cn

[†] Shixian Zhao, Zhou Yao, Qiushi Wang the first three authors contributed equally to this work.

[†]Xueyu Hu and Zhuojing Luo the last two authors contributed equally to this work.

Department of Orthopedics, Xijing Hospital, Air Force Medical University, No. 127 West Changle Road, Shaanxi Province, 710032 Xi'an, China

Supplementary Table S1 Subgroup analysis of VAR in patients with osteoporosis

	LDH Group(n=116)	Control Group(n=92)	P Value
Age（years）	59.79±9.13	59.05±11.06	0.54
Gender , n (%)			0.38
Male	26（22.4）	56（60.9）	
Female	90（77.6）	36（39.1）	
BMI (kg/m^2)	23.88±3.45	24.02±4.68	0.46
VAR(%)	74（63.8）	47（51.1）	0.04*

BMI，Body Mass Index;*,P value＜0.05;VAR, the total prevalence of vertebral axial rotation of lumbar spine

Supplementary Table S2 Subgroup analysis of VAR in patients without osteoporosis

	LDH Group(n=400)	Control Group(n=424)	P Value
Age（years）	45.35±12.49	46.90±10.62	0.44
Gender , n (%)			0.16
Male	214（53.5）	206（48.6）	
Female	186（46.5）	218（51.4）	
BMI (kg/m^2)	23.74±3.46	24.67±4.52	0.39
VAR	191（47.8）	135（31.8）	0.02*

BMI，Body Mass Index;*,P value＜0.05;VAR, the total prevalence of vertebral axial rotation of lumbar spine

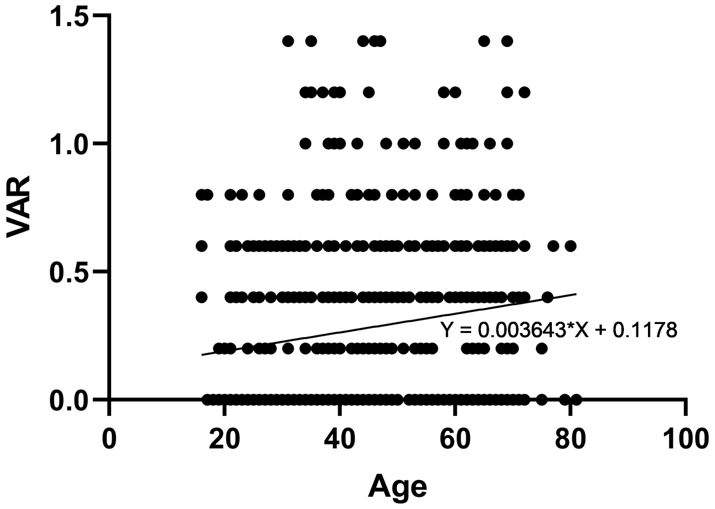

Supplementary Fig S1. Linear correlation analysis between age and VAR in LDH group(r=0.15,P＜0.001).
